# Impact of dyslipidemia and lipid‐lowering therapy with statins in patients with neuroendocrine tumors

**DOI:** 10.1111/jne.13485

**Published:** 2024-12-26

**Authors:** Antongiulio Faggiano, Flaminia Russo, Virginia Zamponi, Franz Sesti, Giulia Puliani, Roberta Modica, Pasqualino Malandrino, Francesco Ferraù, Maria Rinzivillo, Marco Di Muzio, Emanuele Di Simone, Nicolò Panattoni, Pasquale Dolce, Rosa Lauretta, Gianfranco Di Iasi, Antonio Prinzi, Ylenia Alessi, Tiziana Feola, Rossella Mazzilli, Marialuisa Appetecchia, Elisa Giannetta, Francesco Panzuto, Annamaria Colao

**Affiliations:** ^1^ Endocrinology Unit, Department of Clinical and Molecular Medicine, European Neuroendocrine Tumor Society (ENETS) Center of Excellence Sant'Andrea University Hospital, Sapienza University of Rome Rome Italy; ^2^ Department of Experimental Medicine Sapienza University of Rome Rome Italy; ^3^ Oncological Endocrinology Unit IRCCS Regina Elena National Cancer Institute Rome Italy; ^4^ Endocrinology, Diabetology and Andrology Unit, Department of Clinical Medicine and Surgery Federico II University of Naples Naples Italy; ^5^ Endocrinology Unit, Department of Clinical and Experimental Medicine Garibaldi ‐ Nesima Medical Center, University of Catania Catania Italy; ^6^ Department of Human Pathology of Adulthood and Childhood ‘G. Barresi’ University of Messina Messina Italy; ^7^ Digestive Disease Unit, Department of Medical‐Surgical Sciences and Translational Medicine, European Neuroendocrine Tumor Society (ENETS) Center of Excellence Sant'Andrea University Hospital, Sapienza University of Rome Rome Italy; ^8^ Department of Clinical and Molecular Medicine Sapienza University of Rome Rome Italy; ^9^ Department of Public Health and Infectious Diseases Sapienza University of Rome Rome Italy; ^10^ Department of Translational Medical Science Federico II University Naples Italy; ^11^ Department of Biomedical, Dental and Morphological and Functional Imaging Sciences University of Messina Messina Italy; ^12^ Neuroendocrinology Neuromed Institute, IRCCS Pozzilli Italy; ^13^ UNESCO Chair “Education for Health and Sustainable Development” Federico II University Naples Italy

**Keywords:** dyslipidemia, lipid‐lowering therapy, neuroendocrine tumors, statins, tumor progression

## Abstract

Dyslipidemia is a potential unfavorable prognostic factor in neuroendocrine tumors (NETs); conversely, statins proved to have antiproliferative effects in NET cell lines and could be a helpful therapeutic strategy for these patients. The main objective of this observational cohort retrospective study is to explore the associations between dyslipidemia and NET progression and evaluate the potential influence of statins in this context. 393 patients with histologically confirmed gastroenteropancreatic or bronchopulmonary NETs from six Italian centres didicated to NET diagnosis and therapy were included. The cohort included 123 patients with dyslipidemia, 81 of which were taking statins. Clinicopathological data, including patient demographics, tumor characteristics, and treatment details as well as the prevalence, timing of dyslipidemia and hypolipemic therapy were collected. The main outcome measure used is progression‐free survival (PFS). Among the 393 patients, 123 (31.3%) had dyslipidemia. Statins were used by 81 (65.8%) dyslipidemic patients, mostly atorvastatin. Median PFS was 87 months overall, 124 months in non‐dyslipidemic patients, and 72 months in dyslipidemic patients (*p* = .268). Dyslipidemic patients on statins had a significantly better median PFS (108 months) than those not on statins (26 months; *p* = .024). Recurrence‐free survival (RFS) was also evaluated, but no significant differences were found. In conclusion, while PFS was lower in dyslipidemic patients compared to non‐dyslipidemic patients, the difference was not statistically significant. Statin therapy was associated with improved PFS among dyslipidemic patients, suggesting a potential antiproliferative effect of statins in NETs. These findings warrant further investigation to substantiate the role of statins in the management of NETs.

## 1 INTRODUCTION

Neuroendocrine neoplasms (NENs) are a heterogeneous group of epithelial malignancies mainly originating in the gastroenteropancreatic (GEP) and bronchopulmonary (BP) tract.^1^ Their incidence has been steadily rising over the last years.^2^, ^3^ Most NENs are well‐differentiated neuroendocrine tumors (NETs), characterized by low/intermediate proliferation index and commonly slow growth rate. About 30% of them present with metastases at the diagnosis, which varies by site of origin and histotype.^2^ Both the rate of tumor progression in advanced forms and the rate of relapse in the localized ones vary by tumor site and histotype but they remain for the most unpredictable.^4^, ^5^, ^6^, ^7^ Therefore, understanding the biology and behavior of NETs is crucial to define effective therapeutic strategies. A subset of these tumors is associated with genetic syndromes, such as multiple endocrine neoplasia (MEN); however, the great majority of are sporadic and their risk factors are still not completely clear.^1^, ^8^

According to recent studies, dysregulation in lipid metabolism plays an important role in oncogenesis, progression, and altered response to oncological therapies in several types of tumors.^9^, ^10^ Various enzymes involved in fatty acid synthesis are upregulated in numerous types of cancer, such as lung squamous cell cancer, prostate cancer, and melanoma. Through continuous de novo lipogenesis, cancer cells provide themselves with phospholipids, both used for structural and signaling purposes and substitute energy sources, specifically through beta‐oxidation.^10^ By these findings, in the last years, some researchers have pointed out a possible influence of metabolic syndrome (MetS), dyslipidemia, and obesity on NEN development and prognosis,^6^, ^11^, ^12^, ^13^, ^14^ highlighting their role as potential risk factors. The prevalence of dyslipidemia in patients with NETs and its potential impact on tumor progression through lipid metabolism dysregulation highlight an underexplored area in oncology. Lipid metabolism plays a critical role in cancer biology, as tumor cells often reprogram their metabolic pathways to support rapid growth and survival. Dyslipidemia, characterized by elevated levels of cholesterol, triglycerides, or other lipid abnormalities, could contribute to this metabolic reprogramming.

Beta‐oxidation and de novo lipogenesis are fundamental in cancer metabolism, providing energy and essential components for tumor growth and progression. Dyslipidemia, as noted by some authors, may play a pivotal role in NETs by creating a metabolic environment that supports tumor proliferation and resistance to therapy. Elevated lipid levels in dyslipidemia could enhance beta‐oxidation, supplying energy under stress conditions, while promoting lipogenesis to meet the biosynthetic demands of tumor cells. Moreover, the inflammatory and oxidative stress linked to dyslipidemia may facilitate angiogenesis and metastasis, further driving NET progression. Insulin resistance, often associated with dyslipidemia, could amplify lipogenic pathways, exacerbating tumor growth. Modica et al. emphasize the potential of targeting lipid dysregulation as a therapeutic strategy, suggesting that interventions like beta‐oxidation inhibitors or lipogenesis modulators could disrupt the metabolic adaptations critical for NET survival.^10^, ^12^, ^13^

In their retrospective study, Pyo et al. reported higher cholesterol levels in rectal NET patients than in healthy controls.^15^ Similarly, Gallo et al. observed elevated total and LDL cholesterol levels in GEP‐NET patients relative to a control group. They also noted a worsening of clinicopathological characteristics in patients with a higher prevalence of metabolic syndrome, non‐alcoholic fatty liver disease, and visceral adiposity dysfunction.^14^ While definitive data on the role of dyslipidemia in the onset and progression of NETs remain scarce, these findings suggest potential associations between these conditions. This highlights the need for further research, particularly prospective studies, to investigate this relationship in greater detail and establish causal links.

On the other hand, statins, through competitive HMG‐CoA inhibition, have been reported to reduce mevalonate synthesis, farnesylation, and geranylation, causing a decrease in hematic cholesterol levels. At the same time, statins can also reduce proteins involved in tumor proliferation, metastasis, and neo‐angiogenesis; they also have an effect on cell apoptosis through the activation of several caspases.^16^ Since their mechanism of action, in recent years, some studies have tried to use them as possible adjuvant drugs for antineoplastic therapy in different cancer models.^17^ In NET cell lines, specifically atorvastatin and simvastatin decreased proliferation rate, while only simvastatin was able to decrease migration capacity and increase apoptosis.^18^

This observational study aims to explore potential associations between dyslipidemia, statin therapy, and NET progression.

## 2 MATERIALS AND METHODS

### 2.1 Study population

All patients with a histological diagnosis of GEP or BP NETs, in follow‐ups between 2010 and 2023 at six Italian centers dedicated to NET diagnosis and therapy, were included. Clinical–pathological data of all patients and data concerning dyslipidemia (prevalence, timing, type of hypolipemic therapy) were collected and analyzed. Comprehensive data on lipid profiles, as well as information on diabetes mellitus, hypertension, and other components of metabolic syndrome, were not consistently available for all patients, limiting the ability to perform detailed stratification based on lipid parameters. Inclusion criteria were (1) age ≥ 18 years; (2) confirmed histological diagnosis of GEP or BP well‐differentiated G1‐G3 NETs according to World Health Organization (WHO)^19^; (3) ≥12 months follow‐up from NET diagnosis; (4) lipid profile (total cholesterol, HDL [high‐density lipoprotein], LDL [low‐density lipoprotein], triglycerides) evaluated at NET diagnosis. Exclusion criteria were (1) age < 18 years; (2) NETs other than GEP or BP; (3) NEC; (4) follow‐up <12 months; (5) data unavailable.

The study was approved by the Sapienza University Ethic Committee (Reference number 6648/2022) and conducted in accordance with the Declaration of Helsinki. All patients provided written informed consent to data collection.

### 2.2 Study design

This is an observational cohort multicenter‐independent retrospective study. For each patient, demographic features as well as clinical and pathological characteristics (i.e., histology, site of the primary tumor, functionality, tumor stage, tumor grade, genetic syndrome), therapies (surgery and/or systemic therapies) were collected. Progression‐free survival (PFS) and recurrence‐free survival (RFS) were evaluated as outcome indicators. Overall survival was not evaluated because of the low mortality rate.

When dyslipidemia was present, we searched for the time of diagnosis and duration according to NET diagnosis, as well as for treatment with lowering‐lipid agents taken. In particular, statins, resins, omega 3 supplements, ezetimibe, and red rice supplements were considered.

Dyslipidemia was defined as prior or current use of hypolipemic drugs or as abnormally high serum levels of LDL cholesterol or triglycerides, diagnosed through routine hematic tests.^20^, ^21^ Lipid profile was assessed by standard commercial kits. There were no pre‐established time points for the assessment of lipids, except for baseline evaluations.

All the centers participating in the study have managed the patients and scheduled follow‐ups according to the European Neuroendocrine Tumor Society (ENETS) guidelines.^22^

The primary objective of the study was to verify whether dyslipidemia has an impact on clinical outcome (either PFS or RFS). Secondary objectives were (1) to determine the prevalence of lipid metabolism disorders in NET patients and (2) to explore the impact of statins on clinical outcome of tumor progression (PFS and RFS).

### 2.3 Statistical analysis

Assuming that 40% of subjects were suffering from dyslipidemia and 60% were not, with an expected disease progression or death rates of 32% in the non‐dyslipidemic group and 58% in the dyslipidemic group, we calculated that 80 events (progressions or deaths without progression) were needed to achieve a 95% statistical power, with a significance level of *α* = 0.05, and 188 subjects (75 expected in the dyslipidemic group and 113 in the non‐dyslipidemic) detecting a constant hazard ratio (HR) of 0.47 for progressive disease in non‐dyslipidemic patients versus dyslipidemic. Rates of disease progression or death and HR values were derived from Caplin et al., a fundamental NET randomized trial evaluating mPFS in patients treated with somatostatin analogues (SSAs) versus placebo.^23^ With this sample size, assuming that half of the patients with dyslipidemia received statins and the other half did not, we anticipated an 81% power to detect a HR of 0.47 for patients receiving statins compared with those not receiving statins.

Descriptive statistics were obtained using mean ± SD for quantitative variables and frequency (percentage) for categorical variables. Patients were initially categorized into two groups: those suffering by dyslipidemia and those not affected by it. Subsequently, patients in the first group were further subdivided based on whether they were receiving pharmacological treatment with statins. Differences between groups (dyslipidemic vs. non‐dyslipidemic and statin users vs. nonusers) were evaluated using the chi‐square test or Fisher's exact test for categorical variables and the independent samples *t*‐test or Mann–Whitney test for quantitative variables.

Clinical outcome was measured as PFS for patients with locally advanced or metastatic disease undergone to systemic therapy, RFS for patients undergone radical resection. PFS was defined as the time from the first‐line treatment initiation to disease progression (assessed according to clinical practice at the time of diagnosis), last visit, death from any cause, or loss to follow‐up. RFS was defined as the time from radical surgery to disease recurrence (assessed according to clinical practice at the time of diagnosis), last visit, death from any cause, or loss to follow‐up.

The Response Evaluation Criteria in Solid Tumors (RECIST) version 1.1 criteria were used to assess disease progression.^24^ Tumor radiologic assessment was performed by contrast‐enhanced computed tomography (CT) or magnetic resonance (MR) imaging at the time of NET diagnosis and in the follow‐up, after surgery, or during systemic treatment. According to the retrospective design, time points for the radiologic assessment were not preliminarily established. When necessary, gallium‐68 positron emission tomography (68Ga‐PET) scans were also performed. Nevertheless, all the centers followed the ENETS guidelines for the management and timing of patient follow‐ups.^25^, ^26^

Survival analyses were conducted using the Kaplan–Meier method, and median survival times were reported. Univariate Cox proportional hazards regression models were employed to estimate HRs and 95% CIs for the association between dyslipidemia, statin use, and survival outcomes.

Because statin use was found to be significantly associated with PFS in the dyslipidemic group, multivariable analysis was performed using the Cox regression model, adjusting for other factors significantly associated with PFS in the univariate analyses.

All statistical analyses were performed using the R statistical software. The significance level for all statistical tests was set to *α* = 0.05.

## 3 RESULTS

### 3.1 Patient characteristics

**FIGURE 1:**
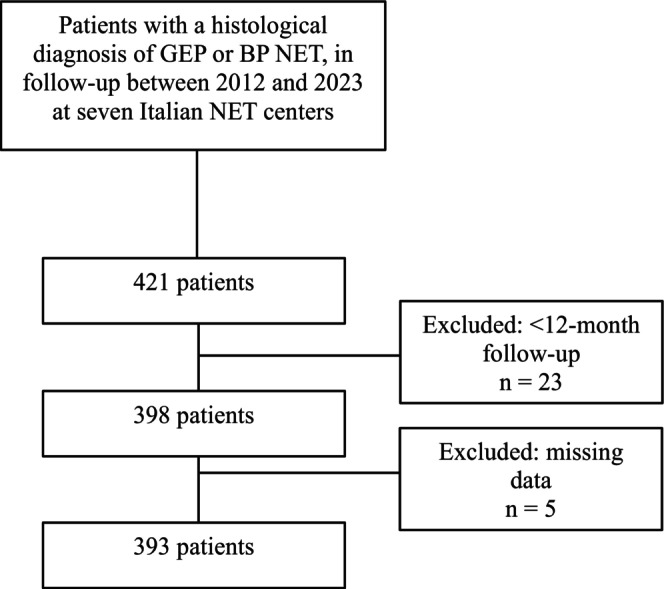
Flowchart for patients considered for the study.

A total of 421 patients were collected; of these, 23 patients were excluded from the study because of short follow‐up time and five because of insufficient data. Finally, 393 patients with a performance status 0–3 according with Eastern Cooperative Oncology Group (ECOG) were considered for the study (Figure 1). Among the whole population, 123 subjects were affected by dyslipidemia (31.3%), while 270 were not (Table 1). There was a homogenous distribution between male and female subjects in the whole population and in subgroups. Mean age was lower in patients not suffering from dyslipidemia than in those affected by it (*p* < .0001). Hereditary NETs were 46 (11.7%), all being related to MEN1. Of them, Seven (5.7%) were in the dyslipidemic and 39 (14.4%) in the non‐dyslipidemic group (*p* < .05). Around 15% of NETs were associated with some types of endocrine syndromes, such as Cushing's (*n*.16), carcinoid (*n*.12), insulinomas (*n*.15), glucagonomas (*n*.2), gastrinomas (*n*.5), or others (*n*.11), without statistically significant differences between the two groups (*p* = .353). In both groups, the highly predominant site of origin was the GEP tract (76.3%), pancreas being the most frequent primary site. Most NETs were G1–G2 (85%) and localized tumor stage (59%).

**TABLE 1:** Characteristics of patients according to dyslipidemia.

Patient characteristics	Total (*N* = 393)	Non‐dyslipidemic (*N* = 270)	Dyslipidemic (*N* = 123)	*p*‐value
Age, mean (SD), years	58.7 (15.0)	56.2 (15.9)	64.2 (11.2)	<.0001
Sex, no. (%)				
Male	196 (49.9)	137 (50.7)	59 (48)	.610
Female	197 (50.1)	133 (49.3)	64 (52)	
BMI, mean (SD), kg/m^2^	26.5 (5.1)	25.9 (5.0)	26.7 (5.4)	.254
Missing, no. (%)	74 (18.8)			
MEN1 syndrome, no. (%)	46 (11.7)	39 (14.4%)	7 (5.7)	.012
Endocrine syndrome, no. (%)	61 (15.5)	45 (16.7)	16 (13.0)	.353
Primary tumor site, no. (%)				
GEP	300 (76.3)	214 (79.2)	6 (69.9)	.048
Bronchial	88 (22.3)	53 (19.6)	35 (28.5)	
Uknown primary	5 (1.2)	3 (1.1)	2 (1.6)	
Tumor stage, no. (%)				
0 (stadio I–II)	231 (58.8)	164 (60.7)	67 (54.5)	.396
1 (stadio III–IV)	126 (32.1)	84 (31.1)	42 (34.1)	
Missing	36 (9.2)	22 (8.1)	14 (11.3)	
Tumor grade, no. (%)				
1	202 (51.4)	145 (53.7)	57 (46.3)	.211
2	132 (33.6)	83 (30.7)	49 (39.8)	
3	22 (5.6)	14 (5.2)	8 (6.5)	
Missing	37 (9.4)	28 (10.4)	9 (7.3)	
Radical surgery, no. (%)	233 (59.3)	155 (57.4)	78 (63.4)	.261
Death, no. (%)	28 (7.1)	18 (6.7)	10 (8.1)	.536

A total of 233 patients (59.3%) underwent radical surgery, while 26 (6.6%) had persistent disease after surgery. Tumor relapse occurred in 87 of 233 (37.3%). As a whole, 209 patients (53.1%) underwent systemic therapy. Overall, the most used first‐line therapy was SSAs (182 patients, 87.1%), followed by chemotherapy (17 patients, 8.1%), radioligand therapy (RLT) (six patients, 2.9%), and targeted therapy (four patients, 1.9%). In 27.1% of cases, more than one line of systemic therapy was required.

In the dyslipidemic group, 41.5% had a diagnosis of dyslipidemia before the NET diagnosis and 49.6% after it, while in the remaining 8.9% of cases, the timing was undetermined. In 26 cases (42.6%) within the subgroup that developed dyslipidemia after the NET diagnosis, the condition occurred concomitantly with systemic therapies, mainly with SSA.

Among the patients with dyslipidemia, 81 (65.8%) were treated with statins, while 26 patients (21.1%) did not receive any hypolipemic therapy. In comparison, 10 patients (8.1%) were on some kind of supplement (mainly fermented red rice or Omega3), four (3.2%) patients were taking fibrates (3.2%), one patient (<1%) was taking ezetimibe, and another one was taking an unspecified hypolipemic drug. Among the 81 patients on statins, 10 of them were taking a combination therapy (statin + ezetimibe/resins). Among statins, the most frequently used was atorvastatin (48.1%), followed by simvastatin (25.9%), rosuvastatin (16.0%), and others unspecified (9.8%).

### 3.2 Clinical outcome

**FIGURE 2:**
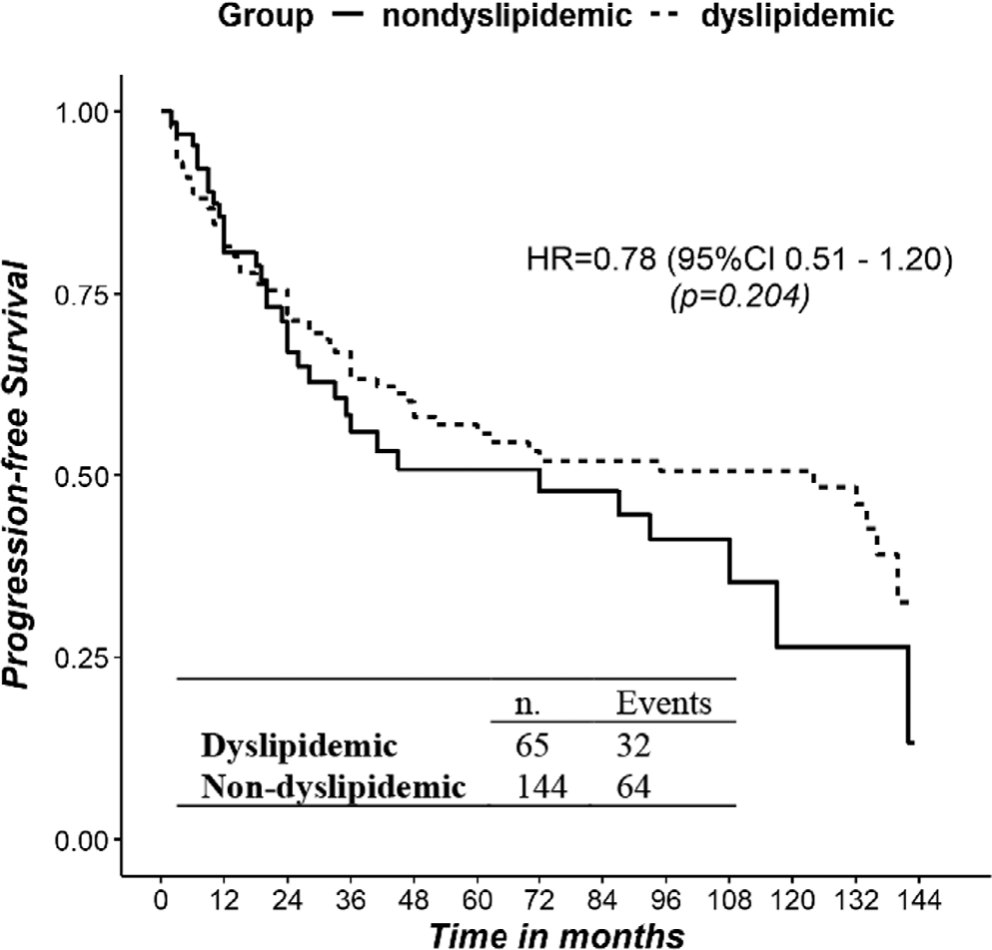
Kaplan–Meier survival curves of PFS between dyslipidemic and non‐dyslipidemic patients. PFS, progression‐free survival.

Estimated median PFS (mPFS) was 87 months in the overall population, 124 months in non‐dyslipidemic, and 72 months in dyslipidemic patients (Figure 2), without significant difference (HR for progression in non‐dyslipidemic vs. dyslipidemic patients was equal to 0.78, 95%CI [0.51; 1.20], *p* = .268).

**FIGURE 3:**
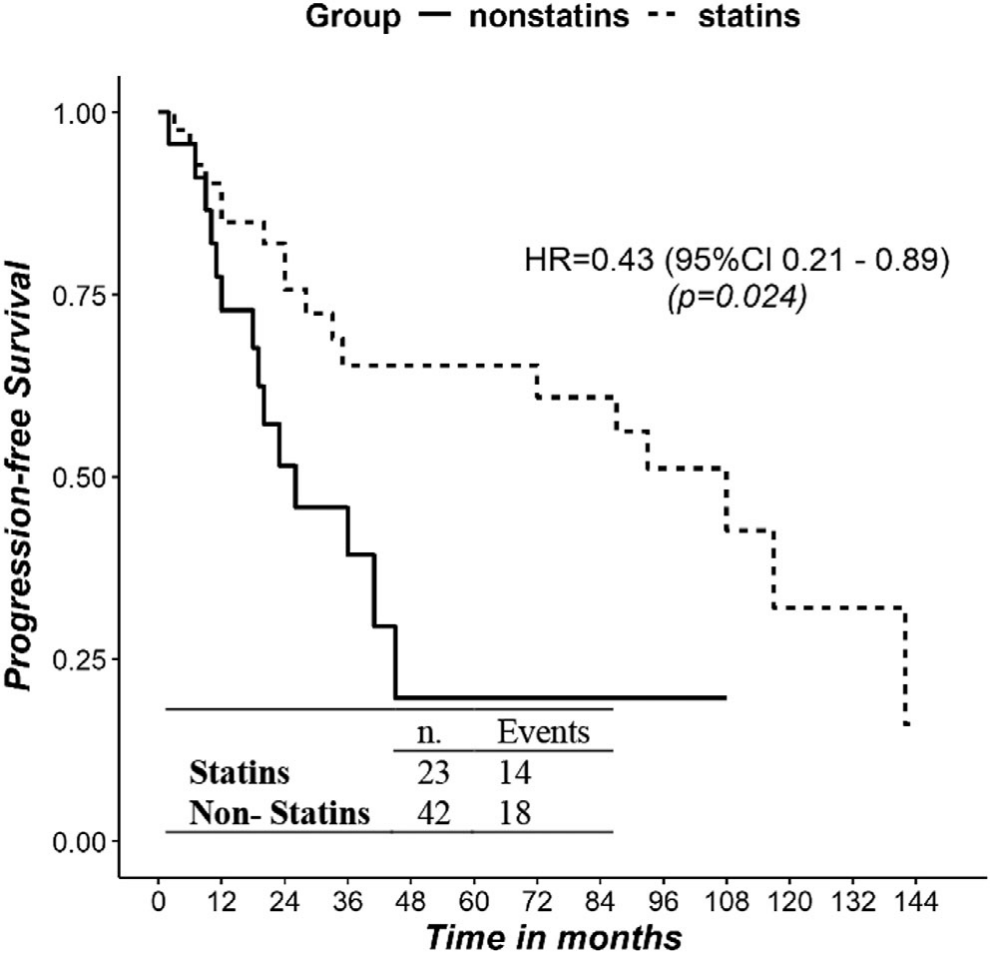
Kaplan–Meier survival curves of PFS between dyslipidemics on statins and dyslipidemics not receiving statins. PFS, progression‐free survival.

In the group with dyslipidemia, mPFS was 26 months in those not taking statins and 108 months in those receiving this treatment (Figure 3). The HR for progression in statin versus non‐statin subgroup was 0.43 ((95% CI 0.21; 0.89), *p* = .024). There was no difference between dyslipidemic patients receiving statins and non‐dyslipidemic patients (HR = 0.98, 95% CI [0.58; 1.66], *p* = .944).

Median RFS (mRFS) was 139 months in the overall population, 143 months in non‐dyslipidemic patients, and 112 months in patients with dyslipidemia, without significant difference (HR for recurrence in non‐dyslipidemic vs. dyslipidemic patients, 0.90, 95% CI [0.58; 1.40], *p* = .637). In the group suffering from dyslipidemia, mRFS was 42 months in those not taking statins and 67 months in those receiving this treatment, without significant difference (HR = 0.86, 95%CI [0.30; 2.49], *p* = .779). Finally, RFS was not significantly different between dyslipidemic patients receiving statins and non‐dyslipidemic patients (HR = 0.89, 95%CI [0.40; 1.97], *p* = .779).

Twenty‐eight patients deceased during the follow‐up. They were mainly advanced stage (82.1%) and high grade (G2 53.6%, G3 42.9%). The mortality rate was not different according to dyslipidemia (Table 1), as well as according to statins (Table 2).

**TABLE 2:** Characteristics of dyslipidemic patients according to therapy with statins.

Patient characteristics	Dyslipidemic (*N* = 123)	Statin (*N* = 81)	Non‐statin (*N* = 42)	*p*‐value
Age, mean (SD), years	64.2 (11.2)	64.0 (11.4)	64.2 (10.9)	.805
Sex, no. (%)				
Male	59 (48)	40 (49.4)	19 (45.2)	.662
Female	64 (52)	41 (50.6)	23 (54.7)	
BMI, mean (SD), kg/m^2^	26.7 (5.4)	26.8 (5.4)	26.6 (5.4)	.749
Missing, no. (%)	30 (24.4)			
MEN1 syndrome, no. (%)	7 (5.7)	7 (8.6)	0 (0)	.049
Endocrine syndrome, no. (%)	16 (13.0)	10 (12.3)	6 (14.2)	.761
Primary tumor site, no. (%)				
GEP	86 (69.9)	56 (69.1)	30 (71.4)	.950
Bronchial	35 (28.5)	23 (28.4)	12 (28.6)	
Unknown primary	2 (1.6)			
Tumor stage, no. (%)				
0 (stadio I–II)	67 (54.5)	47 (58)	20 (47.6)	.523
1 (stadio III–IV)	42 (34.1)	27 (33.3)	15 (35.7)	
Missing	14 (11.4)	7 (8.6)	7 (16.7)	
Tumor grade, no. (%)				
1	57 (46.3)	39 (48.1)	18 (42.8)	.611
2	49 (39.8)	29 (35.8)	20 (47.6)	
3	8 (6.5)	5 (6.2)	3 (7.1)	
Missing	9 (7.3)			
Radical surgery, no. (%)	75 (61)	48 (59.3)	27 (64.3)	.587
Death	10 (8.1)	6 (7.4)	4 (9.5)	.683

Table 3 summarizes the results of the multivariable analysis for PFS in dyslipidemic patients. At the univariate, primary site, tumor grade, and therapy with statins significantly predicted PFS, while tumor grade and therapy with statins remained significant at the multivariable analysis.

**TABLE 3:** Univariate and multivariable analysis for PFS in dyslipidemic patients.

Covariate	Subgroups	Univariate analysis		Multivariable analysis	
		HR (95% CI)	*p*‐value	aHR (95% CI)	*p*‐value
Age	≥60 versus <60 years	0.86 (0.40; 1.78)	.637	/	/
Sex	Female versus male	1.46 (0.70; 3.02)	.309	/	/
Primary site	BP versus GEP	2.82 (1.28; 6.22)	.010	1.80 (0.79; 4.09)	.160
Tumor grade	G2 or G3 versus G1	5.83 (2.03; 16.7)	.001	5.68 (1.81; 15.8)	.002
Therapy with statins	Yes versus no	0.43 (0.21; 0.89)	.024	0.43 (0.19; 0.99)	.047

Abbreviations: BP, bronchopulmonary; GEP, gastroenteropancreatic; PFS, progression‐free survival.

## 4 DISCUSSION

The relationship between cancer and metabolic disorders is well known. Both represent one of the main health issues in the world, affecting millions of people and resulting in high mortality rates.^27^, ^28^ Diabetes mellitus and obesity have been recognized as risk factors for the development and progression of many types of cancer.^9^, ^10^, ^29^, ^30^, ^31^ Also, dyslipidemia, to a lesser extent, appears to be associated with an increased risk of cancer as well as to a more aggressive behavior.^32^, ^33^, ^34^, ^35^ As an observational retrospective study, this investigation does not establish causality but provides valuable preliminary insights that warrant further prospective validation. NENs derive from the diffuse neuroendocrine system and mainly affect the GEP tract and lungs. In the last two decades, these tumors have shown a dramatic increase in incidence, nowadays being estimated at 7 per 100,000.^2^ Diabetes, obesity, and dyslipidemia have been reported as risk factors for many types of NEN, in particular pancreatic and gastrointestinal NETs.^2^, ^6^, ^11^, ^12^ Diabetes was reported to impair PFS in pancreatic NETs, while, on the other hand, the first‐line antidiabetic agent metformin was associated with an improvement of PFS in the same setting.^36^, ^37^ A similar trend appears in the present study for dyslipidemia. Although not statistically significant, patients with dyslipidemia had a much lower mPFS than patients not suffering by it. When the analysis was restricted to the subgroup affected by dyslipidemia, those receiving a therapy with statins had a significantly better mPFS than those not receiving this therapy. At the same time, no difference was found between dyslipidemic patients on statins and non‐dyslipidemics. This difference in PFS persists even when conducting multivariable analysis for other known prognostic factors (age, sex, primary site, grade). This is the first clinical observation of a potential antiproliferative effect of statins in NEN. The lack of significant difference for PFS according to dyslipidemia is reliably due to the low number of progression events, which is expected in NETs because of their slow growth rate. The same picture could be taken in account for RFS rates according to dyslipidemia in patients undergone radical surgery. The impact of statins on PFS, but not RFS, may reflect the differential effects of systemic therapies on advanced versus localized disease. PFS captures the dynamics of tumor progression in advanced stages, where systemic interventions like statins may have a more pronounced influence. However, the relatively short observation period may not be sufficient to draw definitive conclusions, making this hypothesis speculative.

In the last years, some studies, mainly retrospective, focused on the impact of statins on cancer incidence, progression and mortality.^13^, ^38^, ^39^, ^40^, ^41^ This interest derives from the widespread worldwide use of these drugs for dyslipidemia and cardiovascular disease, pathological conditions which frequently overlap cancer. Literature available on this topic regards for the most breast, prostate colorectal, and lung cancer. If no effect has been found on cancer development, on the contrary, the most of studies agree in finding a decrease in both progression and mortality.^17^, ^42^ These effects were observed at a variable extent in all cancer types and were mainly associated to lipophilic statins and initiation of statin therapy before the diagnosis of cancer.^17^

The anticancer activity of statins observed in clinical studies has a well‐defined biological rationale. Statins primarily inhibit the enzyme HMG‐CoA reductase, which has a central role in the biosynthesis of cholesterol. Beyond this, preclinical studies have suggested that these agents may exhibit anticancer effects through various mechanisms, resulting in inhibition of cancer cell proliferation and metastatic spread and induction of apoptosis.^16^, ^43^ One mechanism involves the disruption of cholesterol‐rich lipid rafts in cell membranes, which are crucial for signaling pathways that promote cancer cell growth and survival. Moreover, statins can interfere with the mevalonate pathway, which is pivotal for synthesizing isoprenoids. These molecules play a significant role in the post‐translational modification of proteins like Ras and Rho, which are involved in cell proliferation and survival. By inhibiting these pathways, statins can suppress the growth of cancer cells. In addition to these mechanisms, the gut microbiota has emerged as a key regulator of lipid metabolism and may influence both dyslipidemia and NET progression. Dysbiosis, characterized by an imbalance in the gut microbiota, could alter systemic lipid levels and thereby impact tumor biology, potentially creating an environment conducive to tumor progression.^44^ Future studies should explore these complex interactions, as they may provide novel insights into the interplay between metabolic regulation and cancer biology.

Furthermore, statins have shown the potential to modulate the immune response, target and destroy tumor cells, and reduce inflammation, which is a known risk factor for cancer progression.^45^ Additionally, the LDL receptor has been shown to play a significant role in other tumors by activating key signaling pathways, including MAPK, NF‐κB, and PI3K/Akt, which are critical for tumor growth, survival, and metastasis. This underscores the potential impact of LDL and its receptor in cancer progression and highlights the importance of targeting these pathways in therapeutic strategies.^46^

The present study has some limitations, the main ones being the retrospective design, the low rate of dyslipidemic patients without statins, and the different types of statins. Furthermore, the absence of comprehensive lipid data restricts the ability to discern whether the observed effects of statin therapy are mediated through lipid modulation or independent mechanisms. The lack of systematic documentation of key components of metabolic syndrome, such as diabetes mellitus and hypertension, further restricts the interpretability and generalizability of our findings. Future prospective studies should address these limitations by ensuring a comprehensive and standardized evaluation of lipid profiles and metabolic syndrome variables. On the other hand, the negative impact of the retrospective design is somewhat made less impactful by the participation of two ENETS Centers of excellence in the study and a dedicated multidisciplinary tumor board for NENs in all Centers. The robustness of data quality also derives from the membership of all centers to the Italian Association of NET, sharing common clinical practices for patients' management and data collection. Even with the above‐mentioned limitations, this study reports the first evidence of a potential antiproliferative effect of statins in NET patients, reinforcing recent in vitro observations where different types of these agents (i.e., simvastatin, atorvastatin, lovastatin, rosuvastatin) were able to inhibit proliferation activity and survival in QGP1 cells, while atorvastatin and simvastatin also in BON1 cell lines16.

In conclusion, with the limitations of a retrospective study, these findings highlight for the first time a significant relationship between therapy with statins and improved PFS in patients with BP and GEP advanced NETs affected with dyslipidemia. The lack of significant difference in PFS between dyslipidemic and non‐dyslipidemic patients could also be related to the effects of statins in the formers, attenuating the detrimental impact of dyslipidemia. To establish the cause–effect relationship between statins and NET proliferation, a prospective, phase II, proof‐of‐concept study has been planned at the Sant'Andrea Hospital of Rome.

## AUTHOR CONTRIBUTIONS

**Antongiulio Faggiano:** Writing – original draft; writing – review and editing; supervision; conceptualization; funding acquisition; project administration. **Flaminia Russo:** Writing – original draft; supervision; data curation; investigation. **Virginia Zamponi:** Conceptualization; formal analysis; writing – original draft; writing – review and editing; supervision; data curation. **Franz Sesti:** Supervision; investigation. **Giulia Puliani:** Investigation; supervision. **Roberta Modica:** Investigation; supervision; formal analysis. **Pasqualino Malandrino:** Investigation; supervision. **Francesco Ferraù:** Investigation; supervision. **Maria Rinzivillo:** Investigation; supervision. **Marco Di Muzio:** Formal analysis; supervision. **Emanuele Di Simone:** Formal analysis; supervision. **Nicolò Panattoni:** Formal analysis; supervision. **Pasquale Dolce:** Formal analysis; supervision. **Rosa Lauretta:** Investigation; supervision. **Gianfranco Di Iasi:** Investigation; supervision. **Antonio Prinzi:** Investigation; supervision. **Ylenia Alessi:** Investigation; supervision. **Tiziana Feola:** Investigation; supervision. **Rossella Mazzilli:** Investigation; supervision. **Marialuisa Appetecchia:** Investigation; supervision. **Elisa Giannetta:** Investigation; formal analysis; supervision. **Francesco Panzuto:** Investigation; supervision. **Annamaria Colao:** Investigation; supervision.

## FUNDING INFORMATION

This study was partially supported by a research grant from the Ministry of Health of Italy (T3‐AN‐01).

## CONFLICT OF INTEREST STATEMENT

The authors declare no conflicts of interest.

### PEER REVIEW

The peer review history for this article is available at https://www.webofscience.com/api/gateway/wos/peer-review/10.1111/jne.13485.

## ETHICS STATEMENT

This study was approved by the Sapienza University Ethic Committee (Reference number 6648/2022) and conducted by the Declaration of Helsinki.

## INFORMED CONSENT STATEMENT

All patients provided written informed consent to data collection.

## Data Availability

Data available on request.
